# Compact stars with non-uniform relativistic polytrope

**DOI:** 10.1038/s41598-024-65973-7

**Published:** 2024-07-14

**Authors:** Mohamed I. Nouh, Mona M. Foda, Mohamed S. Aboueisha

**Affiliations:** 1 Astronomy Department, National Research Institute of Astronomy and Geophysics, Helwan, Cairo, 11421 Egypt; 2 Astronomy Department, Faculty of Science, Cairo University, Giza, Egypt

**Keywords:** Composite polytrope, Compact stars, Relativistic effects, General relativity, Mass-radius relation, Astronomy and astrophysics, Astronomy and astrophysics, Statistical physics, thermodynamics and nonlinear dynamics

## Abstract

This paper presents new relativistic composite polytropic models for compact stars by simultaneously solving Einstein field equations with the polytropic state equation to simulate the spherically symmetric, static matter distribution. Using a non-uniform polytropic index, we get the Tolman–Oppenheimer–Volkoff equation for the relativistic composite polytrope (CTOV). To analyze the star's structure, we numerically solve the CTOV equation and compute the Emden and mass functions for various relativistic parameters and polytropic indices appropriate for neutron stars. The calculation results show that, as the relativistic parameter approaches zero, we recover the well-known Lane-Emden equation from the Newtonian theory of polytropic stars; thus, testing the computational code by comparing composite Newtonian models to those in the literature yields good agreement. We compute composite relativistic models for the neutron star candidates Cen X-3, SAXJ1808.4-3658, and PSR J1614-22304. We compare the findings with various existing models in the literature. Based on the accepted models for PSR J1614-22304 and Cen X-3, the star's core radius is predicted to be between 50 and 60% percent of its total radius, while we found that the radius of the core of star SAXJ1808.4-3658 is around 30% of the total radius. Our findings show that the neutron star structure may be approximated by a composite relativistic polytrope, resulting in masses and radii that are quite consistent with observation.

## Introduction

Within the context of the general theory of relativity, research on compact objects and strange stars has emerged as a major topic in theoretical astrophysics in recent years. General relativistic models have been used for fluid spheres with intense gravitational fields, such as neutron stars and strange stars^1–3^. Compact objects (white dwarfs, neutron stars, black holes, and quark stars) are the ultimate stages in the evolution of normal stars. Compact stars are distinguished from high density because nuclear processes cease to exist in their interiors; they cannot support themselves against gravity^4^. The pressure of degenerate gas resists gravity in white dwarfs and neutron stars. The star material is compressed to an infinite density in black holes due to the force of gravity's full dominance over other forces^5^.

Polytropic equations of state have been widely applied to explore the stellar structure and have played an impressive role in astrophysics^6^. This equation of state has been studied in general relativity, for example, by^7–9^. These studies provide an approximate analytical solution for models with different polytropic indices and relativistic parameters. Still, general relativistic polytropes have been thoroughly studied in more compact configurations, such as neutron stars and super Chandrasekhar white dwarfs^7,10–15^.

Most galaxies have stars that develop in gas and dust clouds with a non-uniform matter distribution. Compact stars are isotropic in general; however, isotropy is not a universal property of stellar objects. The extreme interior density and high gravity of compact objects suggest that the pressure within them may not be an ideal fluid. This suggestion implies that the pressure inside the fluid sphere may be split into two nonidentical parts, radial pressure and transverse pressure, which operate in opposite directions^1^. For a relativistic core-envelope model of compact stars, for example^16^, developed an anisotropic core-envelope neutron star model using polytropic index n = 1 for the core layer representing Bose-Einstein condensate matter and polytropic index n = 2 for the envelope layer, which represents the crust. Abellán et al.^9^ provided a generic framework for modeling general relativistic polytropes where both pressures satisfy a polytropic state equation and anisotropic pressure is present. Mathias et al.^17^ generated a core envelope star model in Karmarkar condition where the core is described as a quark matter and the envelope a neutron fluid^18^, generate a charged star model satisfying three layers with distinct equations of state^19^, constructed exact model for a dense stellar object utilizing the Einstein-Maxwell system of equations comprises three interior regions with distinct equations of state, and^20^ established a two-layered model with a quadratic EoS in the envelope layer and a polytropic core. An alternative approach may be called a non-uniform polytrope to model the internal structure of stars and planets (e.g.,^21,22^).

Neutron stars are one of the most compact in the cosmos, named after their core composition of massive neutrons. A neutron star's core crust with diluted neutron matter resembles unitary Fermi gas. Fermion couples with opposite spins behave like bosons at very low temperatures because they have zero total angular momentum. The Pauli exclusion principle does not apply to bosons, unlike fermions. They condense into the lowest-energy single-particle state below a minimal temperature, leading to superfluidity and Bose-Einstein Condensation (BEC)^23^. These stars serve as laboratories for studying high-density nuclear matter using a suitable equation of state (EoS) that relates matter density to pressure. Neutron stars typically have a mass of one to two solar masses (*M* ∼ 1−2 *M*_⊙_) and a radius of around 10−14 km. The mass density (ρ) is around 10^15^ g cm^−3^, which is almost three times the standard nuclear density (ρ_c_) of a heavy atomic nucleus (2.8 × 10^14^ g cm^−3^)^24–27^.

In astrophysics, composite polytropic models are occasionally employed to simulate the interior structure of stars where equilibrium conditions vary across different zones^31^. Among the examples are stars in the late stages of evolution (e.g., white dwarfs and neutron stars) and stars with radiative envelopes around convective cores, or vice versa. Matter in neutron stars can be as diverse as nuclei buried in a sea of electrons at low densities in the outer crust, structures in the inner crust that are becoming more and more neutron-rich, uniform matter in the outer core that is extremely neutron-rich, and potentially exotic states of matter at high densities in the inner core. As a result, the double-layered hypothesis (core-envelope), which considers two neighboring layers of the star with distinct matter distributions and macroscopic physical characteristics, starts to make sense^16,32^.

The Tolman–Oppenheimer–Volkoff (TOV) equation constrains the structure of spherically symmetric objects of isotropic material in static gravitational equilibrium, as modeled by general relativity. Besides applying the TOV equation to compact stars, there are many applications in astrophysics; for example, Gupta et al.^28^ determined the hydrostatic masses of the Galaxy cluster observed using Chandra X-ray data. Inspired by the new findings of star motions under the influence of dark matter, Bors and Stańczy^29^ explore the model representing the interaction of relativistic gravitationally attractive diffusive fermionic particles evaporating at high energy clusters of stellar systems^30^.

In the present paper, we derive the composite TOV equations (CTOV) with a non-uniform polytropic index to model condensed matter in compact stars. We numerically integrate the CTOV equations for a wide range of the polytropic index and relativistic parameters. We will compute composite polytropic models with n=1 and n=2 for neutron stars. The structure of the paper is as follows: in “Formulation of CTOV equation”, we derived the CTOV equation, “Results” deals with the results, and “Conclusion” is devoted to the conclusion.

## Formulation of CTOV equation

The line element describing the interior space–time of a static spherically symmetric star in standard coordinates $x^{a} = \left( {t,r,\theta ,\phi } \right)$, takes the following form^7^

1 $$ ds^{2} = e^{2\nu \left( r \right)} c^{2} dt^{2} - e^{2\lambda \left( r \right)} dr^{2} - r^{2} \left( {d\theta^{2} + \sin^{2} \theta d\phi^{2} } \right), $$

where $\nu$ and $\lambda$ are functions of $r$ only. Assuming that the matter distribution within the star is isotropic with the energy–momentum tensor in the form

2 $$ T_{\alpha \beta } = \rho u_{\alpha } u_{\beta } + \frac{P}{{c^{2} }}\left( {u_{\alpha } u_{\beta } - g_{\alpha \beta } } \right), $$

where $\rho$ is the mass density, $P$ is the isotropic pressure and $u_{\alpha }$ is the four-velocity vector satisfying the condition $u_{\alpha } u_{\beta } = 1$. The Einstein field equations for the metric Eq. (1) and energy–momentum tensor Eq. (2) can be written as

3 $$ e^{ - 2\lambda } \left( {\frac{{2\lambda^{\prime}}}{r} - \frac{1}{{r^{2} }}} \right) + \frac{1}{{r^{2} }} = \frac{8\pi G}{{c^{2} }}\rho , $$

4 $$ e^{ - 2\lambda } (\frac{1}{{r^{2} }} + \frac{{2v^{\prime}}}{r}) - \frac{1}{{r^{2} }} = \frac{8\pi G}{{c^{4} }}P, $$

5 $$ e^{ - 2\lambda } \left( {\nu^{\prime\prime} + \nu^{{\prime}{2}} - \nu^{\prime}\lambda^{\prime} + \frac{1}{r}\left( {\nu^{\prime} - \lambda^{\prime}} \right)} \right) = \frac{8\pi G}{{c^{4} }}P, $$

where $G$ is the gravitational constant and $c$ is the speed of light.

Using the radial component of the conservation law of energy–momentum tensor ($T^{\alpha \beta }_{;\beta } = 0$) one can obtain the following relation

6 $$ \left( {\rho c^{2} + P} \right)\nu^{\prime}\left( r \right) = - P^{\prime}\left( r \right), $$

From Eq. (3) we get

7 $$ e^{2\lambda } = \left( {1 - \frac{2G\,m(r)}{{c^{2} r}}} \right)^{ - 1} , $$

where $m(r)$ is the gravitational mass enclosed in a sphere with radius $r$ and is given by

8 $$ m(r) = \int\limits_{0}^{r} {4\pi \,r^{{\prime}{2}} \rho dr} . $$

The first derivative of the mass function can be obtained from Eq. (4) as

9 $$ \nu^{\prime}\left( r \right) = \frac{{\frac{G}{{c^{2} }}m(r) + \frac{4\pi G}{{c^{4} }}Pr^{3} }}{{r\left( {r - \frac{2G}{{c^{2} }}m(r)} \right)}}. $$

By inserting Eq. (8) in Eq. (6), the TOV-equation can be written as

10 $$ P^{\prime}\left( r \right) = - \left( {\rho c^{2} + P} \right)\frac{{\frac{G}{{c^{2} }}m(r) + \frac{4\pi G}{{c^{4} }}Pr^{3} }}{{r\left( {r - \frac{2G}{{c^{2} }}m(r)} \right)}}. $$

Imposing the pressure *P* is related to the density $\rho$ in the form of the polytropic equation of state as

11 $$ P = k\rho^{{1 + \frac{1}{n}}} , $$

where

12 $$ \rho = \rho_{c} \theta^{n} , $$

where $\rho_{c}$ is the central density, $k$ is the pressure constant, and $\theta$ is the Emden function (the ratio of the density to the central density $\rho /\rho_{c}$). The polytropic index is denoted by $n$, which is given by the non-uniform formula adopted by^31^ as

13 $$ n = a - b\tanh \left( {\frac{{q - q_{1} }}{\epsilon}} \right), $$

where *a* and *b* are determined according to the physical situation of the stellar internal structure (e.g., the star with radiative core and convective envelope), where $q_{1} = r_{0} /R$ denotes the core boundary, $q = r/R$, and $\epsilon$ is the width of the transition layer.

According to the definition of the variable polytropic index, Eq. (13), *n* might take any possible values between the polytropic indexes n_1_ and n_2_ (where n_1_ works for the region from the center of the star to the first boundary of the transition region determined by $\epsilon$ while n_2_ works from the transition region's second boundary to the star's surface). From Eq. (13), as *q* takes values between 0–1, the polytropic indexes take values between n_1_ and n_2_. As a result, the star is modeled by a composite polytrope, not a double polytrope. For example, Wei^31^ takes n_1_ = 4, n_2_ = 1.5, a = 2.75, b = 1.25, q_1_ = 0.7, and $\epsilon$ = 0.001 to model the internal structure of the sun.

Performing the first derivative to Eq. (11) gives

14 $$ \frac{dP}{{dr}} = k\theta^{n + 1} \frac{{d\rho_{c}^{{1 + \frac{1}{n}}} }}{dr} + k\rho_{c}^{{1 + \frac{1}{n}}} \frac{{d\theta^{n + 1} }}{dr}. $$

To obtain $\frac{{d\rho_{c}^{{1 + \frac{1}{n}}} }}{dr}$, we let

15 $$ y = \rho_{c}^{{1 + \frac{1}{n}}} , $$

then

16 $$ \ln \left( y \right) = \left( {1 + \frac{1}{n}} \right)\ln \left( {\rho_{c} } \right). $$

Differentiate both sides, we get

17 $$ \frac{1}{y}\frac{dy}{{dr}} = \frac{{ - n^{\prime}}}{{n^{2} }}\ln \left( {\rho_{c} } \right), $$

where

18 $$ n^{\prime} = - \frac{b}{{R\epsilon}}{\text{sech}}^{2} \left( {\frac{{q - q_{1} }}{\epsilon}} \right). $$

Now, using Eq. (17), we can write

19 $$ \frac{{d\rho_{c}^{{1 + \frac{1}{n}}} }}{dr} = \frac{a}{{n^{2} R\epsilon}}\rho_{c}^{{1 + \frac{1}{n}}} \ln \left( {\rho_{c} } \right)\,{\text{sech}}^{2} \left( {\frac{{q - q_{1} }}{\epsilon}} \right). $$

To calculate $\frac{{d\theta^{n + 1} }}{dr}$, we let

20 $$ Z = \theta^{n + 1} , $$

then

21 $$ \ln \left( Z \right) = \left( {n + 1} \right)\ln \left( \theta \right), $$

differentiate both sides, we get

22 $$ \frac{1}{Z}Z^{\prime} = n^{\prime}\ln \left( \theta \right) + \frac{n + 1}{\theta }\theta^{\prime}. $$

Substitute Eq. (18) into Eq. (22), we get

23 $$ \frac{1}{Z}Z^{\prime} = - \frac{b}{{R\epsilon}}{\text{sech}}^{2} \left( {\frac{{q - q_{1} }}{\epsilon}} \right)\ln \left( \theta \right) + \frac{n + 1}{\theta }\theta^{\prime}, $$

then

24 $$ \frac{{d\theta^{n + 1} }}{dr} = - \frac{b}{{R\epsilon}}{\text{sech}}^{2} \left( {\frac{{q - q_{1} }}{\epsilon}} \right)\theta^{n + 1} \ln \left( \theta \right) + \frac{n + 1}{\theta }\theta^{n + 1} \theta^{\prime}. $$

Substitute Eqs. (19) and (24) into Eq. (14), we obtain

25 $$ \begin{gathered} \frac{dP}{{dr}} = \frac{b}{{n^{2} R\epsilon}}k\theta^{n + 1} \rho_{c}^{{1 + \frac{1}{n}}} \ln \left( {\rho_{c} } \right)\,{\text{sech}}^{2} \left( {\frac{{q - q_{1} }}{\epsilon}} \right) \hfill \\ \,\,\,\,\,\,\,\,\, + \left( {n + 1} \right)k\rho_{c}^{{1 + \frac{1}{n}}} \theta^{n} \theta^{\prime} - \frac{b}{{R\epsilon}}k\rho_{c}^{{1 + \frac{1}{n}}} {\text{sech}}^{2} \left( {\frac{{q - q_{1} }}{\epsilon}} \right)\theta^{n + 1} \ln \left( \theta \right), \hfill \\ \end{gathered} $$

Equation (25) shows that the constant *a* disappeared due to the differentiation of Eq. (13); as a result, the constant *b* is only found in Equation (25).

Then, we can rewrite Eq. (25) as

26 $$ \frac{dP}{{dr}} = \frac{b}{{R\epsilon}}P\,{\text{sech}}^{2} \left( {\frac{{q - q_{1} }}{\epsilon}} \right)\left( {\frac{1}{{n^{2} }}\ln \left( {\rho_{c} } \right) - \ln \left( \theta \right)} \right) + \left( {n + 1} \right)\frac{P}{\theta }\theta^{\prime}\left( r \right), $$

Inserting Eqs. (11), (12), and (26) into Eq. (6) yields

27 $$ \begin{gathered} \left( {\rho_{c} \theta^{n} c^{2} + k\rho_{c}^{{1 + \frac{1}{n}}} \theta^{1 + n} } \right)\frac{d\nu }{{dr}} = \hfill \\ \, - \frac{b}{{R\epsilon}}k\rho_{c}^{{1 + \frac{1}{n}}} \theta^{1 + n} \,{\text{sech}}^{2} \left( {\frac{{q - q_{1} }}{\epsilon}} \right)\left( {\frac{1}{{n^{2} }}\ln \left( {\rho_{c} } \right) - \ln \left( \theta \right)} \right) - \left( {n + 1} \right)k\rho_{c}^{{1 + \frac{1}{n}}} \theta^{n} \theta^{\prime}\left( r \right), \hfill \\ \end{gathered} $$

then

28 $$ \left( {1 + \frac{{k\rho_{c}^{\frac{1}{n}} }}{{c^{2} }}\theta } \right)\frac{d\nu }{{dr}} = - \frac{b}{{R\epsilon}}\frac{{k\rho_{c}^{\frac{1}{n}} }}{{c^{2} }}\theta \,{\text{sech}}^{2} \left( {\frac{{q - q_{1} }}{\epsilon}} \right)\left( {\frac{1}{{n^{2} }}\ln \left( {\rho_{c} } \right) - \ln \left( \theta \right)} \right) - \left( {n + 1} \right)\frac{{k\rho_{c}^{\frac{1}{n}} }}{{c^{2} }}\theta^{\prime}\left( r \right), $$

where the relativistic parameter $\sigma$ is given by

29 $$ \sigma = \frac{{P_{c} }}{{\rho_{c} c^{2} }} = \frac{{k\rho_{c}^{\frac{1}{n}} }}{{c^{2} }}. $$

After some manipulations, Eq. (28) can be written as

30 $$ \frac{d\nu }{{dr}} = - \frac{b}{{R\epsilon}}\frac{\sigma \, \theta }{{\left( {1 + \sigma \, \theta } \right)}}\,{\text{sech}}^{2} \left( {\frac{{q - q_{1} }}{\epsilon}} \right)\left( {\frac{1}{{n^{2} }}\ln \left( {\rho_{c} } \right) - \ln \left( \theta \right)} \right) - \frac{{\left( {n + 1} \right)\sigma \, \theta^{\prime}}}{{\left( {1 + \sigma \, \theta } \right)}}, $$

Inserting Eqs. (29), (30) and (7) into Eq. (4), we get

31 $$ \begin{gathered} \frac{1}{{r^{2} }}\left( {1 - \frac{2G\,m(r)}{{c^{2} r}}} \right)\left( {1 - \frac{2br}{{R\epsilon}}\frac{\sigma \, \theta }{{\left( {1 + \sigma \, \theta } \right)}}\,{\text{sech}}^{2} \left( {\frac{{q - q_{1} }}{\epsilon}} \right)\left( {\frac{1}{{n^{2} }}\ln \left( {\rho_{c} } \right) - \ln \left( \theta \right)} \right) - \frac{{2\left( {n + 1} \right)\sigma r}}{{\left( {1 + \sigma \, \theta } \right)}}\theta^{\prime}} \right)\, \hfill \\ \,\,\,\,\,\,\,\,\,\,\,\,\,\,\,\,\,\,\, - \frac{1}{{r^{2} }} - \frac{8\pi G}{{c^{4} }}k\rho_{c}^{{1 + \frac{1}{n}}} \theta^{n + 1} = 0\,\,\,\,\, \hfill \\ \end{gathered} $$

Multiply the last equation by $r^{2}$ we obtain

32 $$ \left( {1 - \frac{2G\,m(r)}{{c^{2} r}}} \right)\left( {1 - \frac{2br}{{R\epsilon}}\frac{\sigma \, \theta }{{\left( {1 + \sigma \, \theta } \right)}}\,{\text{sech}}^{2} \left( {\frac{{q - q_{1} }}{\epsilon}} \right)\left( {\frac{1}{{n^{2} }}\ln \left( {\rho_{c} } \right) - \ln \left( \theta \right)} \right) - \frac{{2\left( {n + 1} \right)\sigma r}}{{\left( {1 + \sigma \, \theta } \right)}}\theta^{\prime}} \right)\,\, - 1 - \frac{2G}{{c^{4} }}k\rho_{c}^{\frac{1}{n}} \theta \frac{dm}{{dr}} = 0\,, $$

where we use

33 $$ \frac{dm\left( r \right)}{{dr}} = 4\pi r^{2} \rho_{c} \theta^{n} . $$

Using Eq. (29), we get

34 $$ \begin{gathered} \left( {1 - \frac{2G\,m(r)}{{c^{2} r}}} \right)\left( {1 - \frac{2br}{{R\epsilon}}\frac{\sigma \, \theta }{{\left( {1 + \sigma \, \theta } \right)}}\,{\text{sech}}^{2} \left( {\frac{{q - q_{1} }}{\epsilon}} \right)\left( {\frac{1}{{n^{2} }}\ln \left( {\rho_{c} } \right) - \ln \left( \theta \right)} \right)} \right)\, \hfill \\ \,\,\,\,\,\,\,\,\,\,\,\,\,\,\,\,\,\,\, - \frac{{2\left( {n + 1} \right)\sigma \, r}}{{\left( {1 + \sigma \, \theta } \right)}}\left( {1 - \frac{2G\,m(r)}{{c^{2} r}}} \right)\theta^{\prime} - 1 - \frac{2G}{{c^{2} }}\sigma \, \theta \frac{dm}{{dr}} = 0\,,\,\,\,\, \hfill \\ \end{gathered} $$

Now, we can rewrite Eq. (34) as

35 $$ \begin{gathered} \frac{{\sigma \left( {n + 1} \right)r}}{{\left( {1 + \sigma \, \theta } \right)}}\left( {1 - \frac{2G\,m(r)}{{c^{2} r}}} \right)\theta^{\prime} + \frac{G\,m(r)}{{c^{2} r}} + \frac{br}{{R\epsilon}}\frac{\sigma \, \theta }{{\left( {1 + \sigma \, \theta } \right)}}\,{\text{sech}}^{2} \left( {\frac{{q - q_{1} }}{\epsilon}} \right)\left( {\frac{1}{{n^{2} }}\ln \left( {\rho_{c} } \right) - \ln \left( \theta \right)} \right)\left( {1 - \frac{2G\,m(r)}{{c^{2} r}}} \right)\, \hfill \\ \,\,\,\,\,\,\,\, + \,\frac{G}{{c^{2} }}\sigma \, \theta \frac{dm}{{dr}} = 0\,\,\,\, \hfill \\ \end{gathered} $$

Let

36 $$ r = \frac{\xi }{l},\,\,\,\,\,\upsilon = \frac{m}{\rm M},\,\,\,\,\,l = \left( {\frac{{4\pi G\rho_{c} }}{{\sigma (n + 1)c^{2} }}} \right)^{1/2} , $$

where $M$ and $l$ are the characteristic mass scale and characteristic length scale of the polytrope.

Using Eq. (36), Eq. (35) can be written as

37 $$ \begin{gathered} \frac{{\sigma \left( {n + 1} \right)}}{{\left( {1 + \sigma \, \theta } \right)}}\left( {1 - \frac{{2G\, \, \upsilon \, {\rm M} \, l}}{{c^{2} \xi }}} \right)\xi \frac{d\theta }{{d\xi }} + \frac{{G \, \,\upsilon \, {\rm M} \, l}}{{c^{2} \xi }} \hfill \\ + \frac{b\xi }{{lR}}\frac{\sigma \, \theta }{{\epsilon\left( {1 + \sigma \, \theta } \right)}}\,{\text{sech}}^{2} \left( {\frac{{q - q_{1} }}{\epsilon}} \right)\left( {\frac{{\ln \left( {\rho_{c} } \right)}}{{n^{2} }} - \ln \left( \theta \right)} \right)\left( {1 - \frac{{2G\, \, \upsilon \, {\rm M} \, l}}{{c^{2} \xi }}} \right)\, \hfill \\ \,\,\,\,\,\,\,\, + \,\frac{G}{{c^{2} }}\sigma \, \theta \, {\rm M} \, l\frac{d\upsilon }{{d\xi }} = 0\,\,\,\, \hfill \\ \end{gathered} $$

Knowing that

38 $$ {\rm M} = \frac{{4\pi \rho_{c} }}{{l^{3} }}\upsilon = \frac{{c^{2} \left( {1 + n} \right)\sigma \upsilon }}{Gl},\,\,\,\,\,\,\,\,\,\,\,\,\,\,\,\,\,\,\,\,\,\xi_{1} = lR, $$

then Eq. (37) can be written as

39 $$ \begin{gathered} \frac{{\xi^{2} }}{{\left( {1 + \sigma \theta } \right)}}\left( {1 - \frac{{2\sigma \left( {n + 1} \right)\upsilon }}{\xi }} \right)\frac{d\theta }{{d\xi }} \hfill \\ + \frac{{b\xi^{2} }}{{\xi_{1} }}\frac{\theta }{{\epsilon\left( {1 + \sigma \theta } \right)\left( {n + 1} \right)}}\,{\text{sech}}^{2} \left( {\frac{{q - q_{1} }}{\epsilon}} \right)\left( {\frac{{\ln \left( {\rho_{c} } \right)}}{{n^{2} }} - \ln \left( \theta \right)} \right)\left( {1 - \frac{{2\sigma \left( {n + 1} \right)\upsilon }}{\xi }} \right)\, \hfill \\ \,\,\,\,\,\,\,\, + \upsilon + \,\sigma \theta \xi \frac{d\upsilon }{{d\xi }} = 0\,\,\,\, \hfill \\ \end{gathered} $$

and

40 $$ \upsilon^{\prime}\left( \xi \right) = \xi^{2} \theta^{n} \left( \xi \right) $$

where the Eqs. (39) and (40) must be solved under the boundary conditions

41 $$ \theta \left( 0 \right) = 1,\,\,\,\,\,\,\,\,\,\,\,\,\,\,\,\,\,\,\,\,\upsilon \left( 0 \right) = 0 $$

## Results

As we see from Eqs. (11), (12), and (39), the physical parameters of the polytrope are functions of the Emden and mass functions ($\theta$,$\nu$).To calculate the Emden and the mass functions ($\theta$,$\nu$), we numerically integrated Eqs. (28) using the Rung-Kutta method package of Mathematica 13.2. The inputs to the code are the relativistic parameter (σ), the core boundary (q_1_), the ratio q, and the width of the transition layer ($\epsilon$).The appropriate values of the constants *a* and *b* in Eq. (13) could be determined from the two relations *a* = (n_1_ + n_2_)/2 and b = n_1_-*a*, where n_1_ and n_2_ are the polytopic indices of the first and second polytropes, respectively. In Table 1, we listed some of the possible values of *a*, *b*, n_1_, and n_2_. We can compute the model with a single polytrope if we use n_1_ = n_2_ (i.e., a = n and b = 0).

**Table 1:** The constants *a* and *b* in Eq. (13) and the corresponding polytropic indices of the first polytrope n_1_ and the second polytrope n_2_.

n_1_	n_2_	*A*	*B*
0	1	0.5	−0.5
0.5	1	0.75	−0.25
1	2	1.5	−0.5
2	1	1.5	0.5
3	1.5	2.25	0.75
3	3	3	0
4	1.5	2.75	1.25

**Figure 1:**
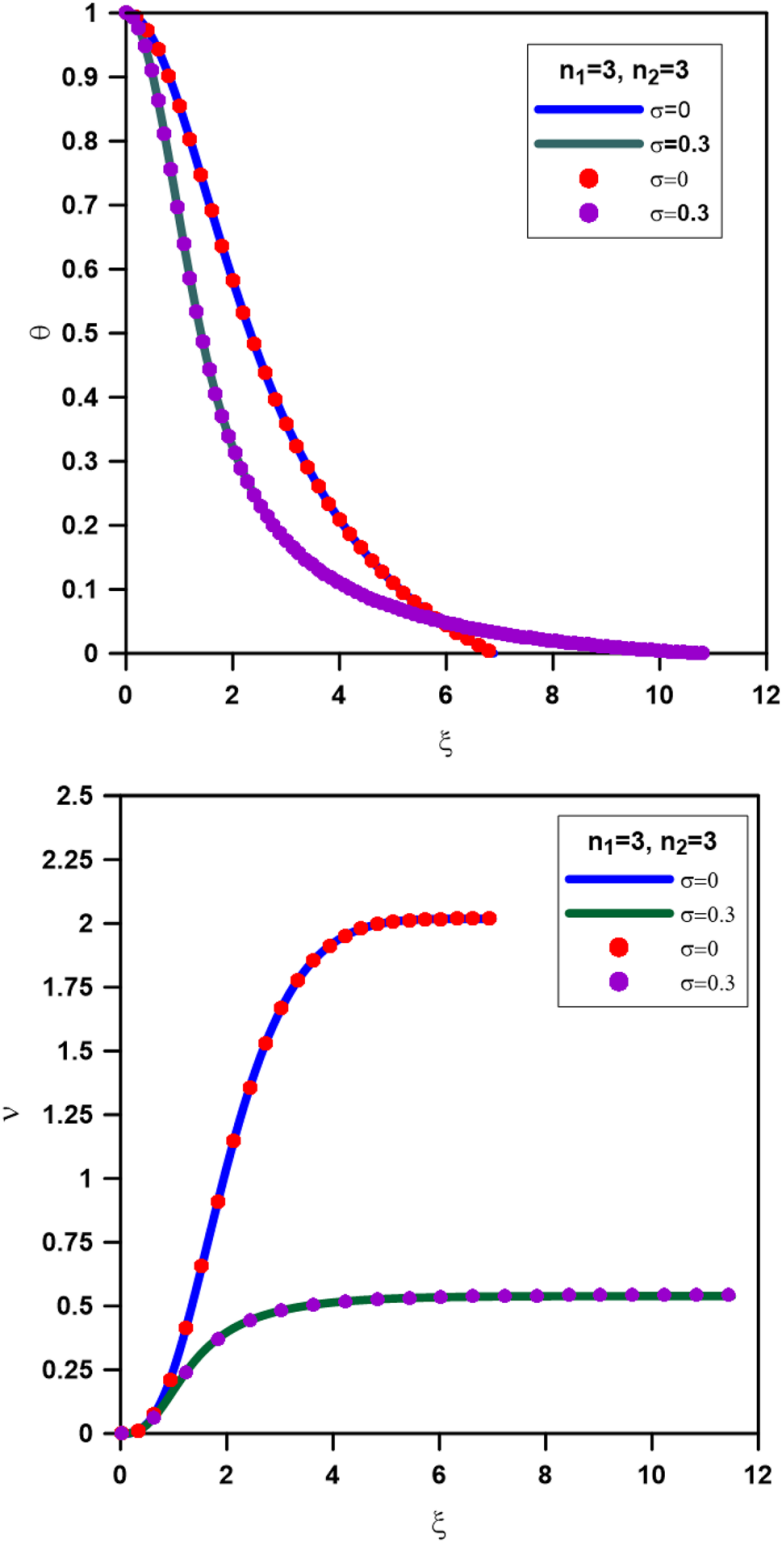
Comparison between solutions of composite polytrope (n_1_ = 3 and n_2_ = 3, CTOV equation-solid lines) and single polytrope (n = 3, TOV equation-dashed lines) with n = 3, σ = 0 and 0.3. The upper panel is for the Emden function, and the lower panel is for the mass function.

We test the code by performing different calculations: the first test is for the Newtonian and relativistic single polytrope with n = 3 (i.e., n_1_ = 3, n_2_ = 3, *a* = 3, *b* = 0). In Fig. 1, we plot the Emden (the upper panel) and mass (the lower panel) functions calculated with the relativistic parameters $\sigma = 0$ for Newtonian polytrope and $\sigma = 0.3$ for relativistic polytropes. For comparison, the calculations for the single Newtonian and relativistic polytropes are taken from^8^, which indicates that we agreed with maximum absolute errors of 10^–7^ and 10^–5^ for the Newtonian and relativistic polytropes, respectively.

**Figure 2:**
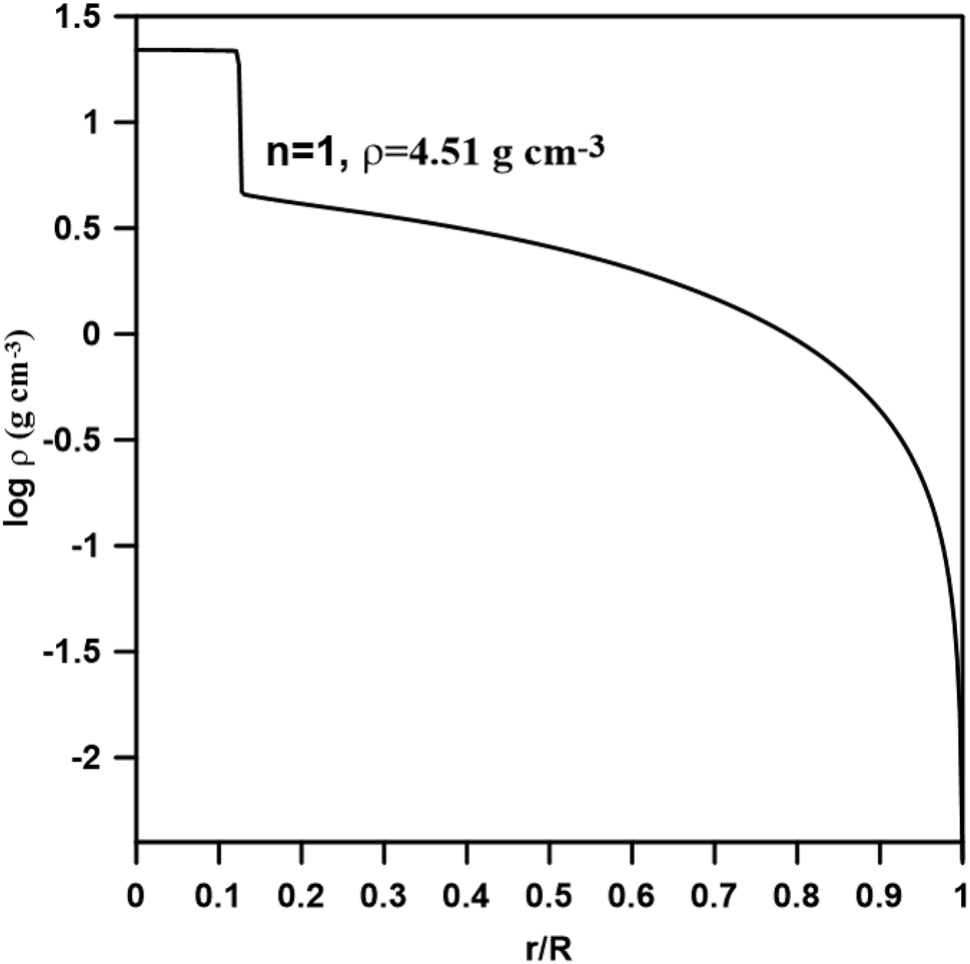
Composite polytropic model for the planet with 10 M _Θ_, n_1_ = 0.5 and n_2_ = 1, central density 22.79 g cm^−3^.

Now, we shall move to the calculation of composite Newtonian polytrope (i.e. $\sigma = 0$, n_1_ to n_2_). We calculated a model appropriate for the planet with a mass of 10 M _Θ_ (M _Θ_ is the mass of the earth), n_1_ = 0.5, n_2_ = 1, and a central density of 22.79 g cm^−3^^30^. Figure 2 displays the density profile of the planet model. The calculation revealed that the density at the n_1_–n_2_ interface is 4.51 g cm^−3^, which agrees with the interface density (0.4.47 g cm^−3^) calculated by^21^.

A neutron star (NS) interior comprises two primary components: the solid crust and liquid core. Nuclear clusters comprise the non-uniform crust, whereas the core is homogenous and uniform. We calculated composite polytropic models using n_1_ = 1 and n_2_ = 2 with two values of the central pressures $P_{c} = 1 \times 10^{35}$ and $2 \times 10^{35}$ dyne cm^−2^ and different central densities, as listed in Table 2. The relativistic parameter is calculated from the equation $\sigma = P_{c} /(\rho_{c} {\text{ c}}^{2} )$; the maximum value of σ that fulfils the causality condition is given by σ _max_ = n/(n + 1), where the sound velocity (v_s_) must be smaller than the speed of light (v_s_/c < 1). It is important to mention that the interface of the present composite models occurs when the polytropic index changes at the interface from n = 1 to n = 1.00001; this indicates that the models are of multi-layered polytrope, not double-layered polytrope.

**Table 2:** Values of the central densities, central pressures, and relativistic parameters for eight composite models calculated with polytropic indexes n_1_ = 1 to n_2_ = 2.

$P_{c} = 1 \times 10^{35} {\text{ (dyne cm}}^{ - 2} {)}$						$P_{c} = 2 \times 10^{35} {\text{ (dyne cm}}^{ - 2} {)}$					
models	$\rho_{c} (10^{15} )$ $({\text{g cm}}^{ - 3} )$	$\sigma$	*R* (km)	*M* (*M*_ʘ_)	CF	models	$\rho_{c} (10^{15} )$ $({\text{g cm}}^{ - 3} )$	$\sigma$	*R* (km)	*M* (*M*_ʘ_)	CF
Model 1	1	0.1111	8.7969	0.7848	0.090	Model 5	1	0.2222	12.0391	1.7721	0.147
Model 2	1.5	0.0744	5.8619	0.3658	0.062	Model 6	1.5	0.1481	8.1546	0.9056	0.111
Model 3	2	0.0555	4.3964	0.2106	0.048	Model 7	2	0.1111	6.2230	0.5542	0.090
Model 4	2.5	0.0444	3.5171	0.1366	0.038	Model 8	2.5	0.0888	4.4740	0.3649	0.073

**Figure 3:**
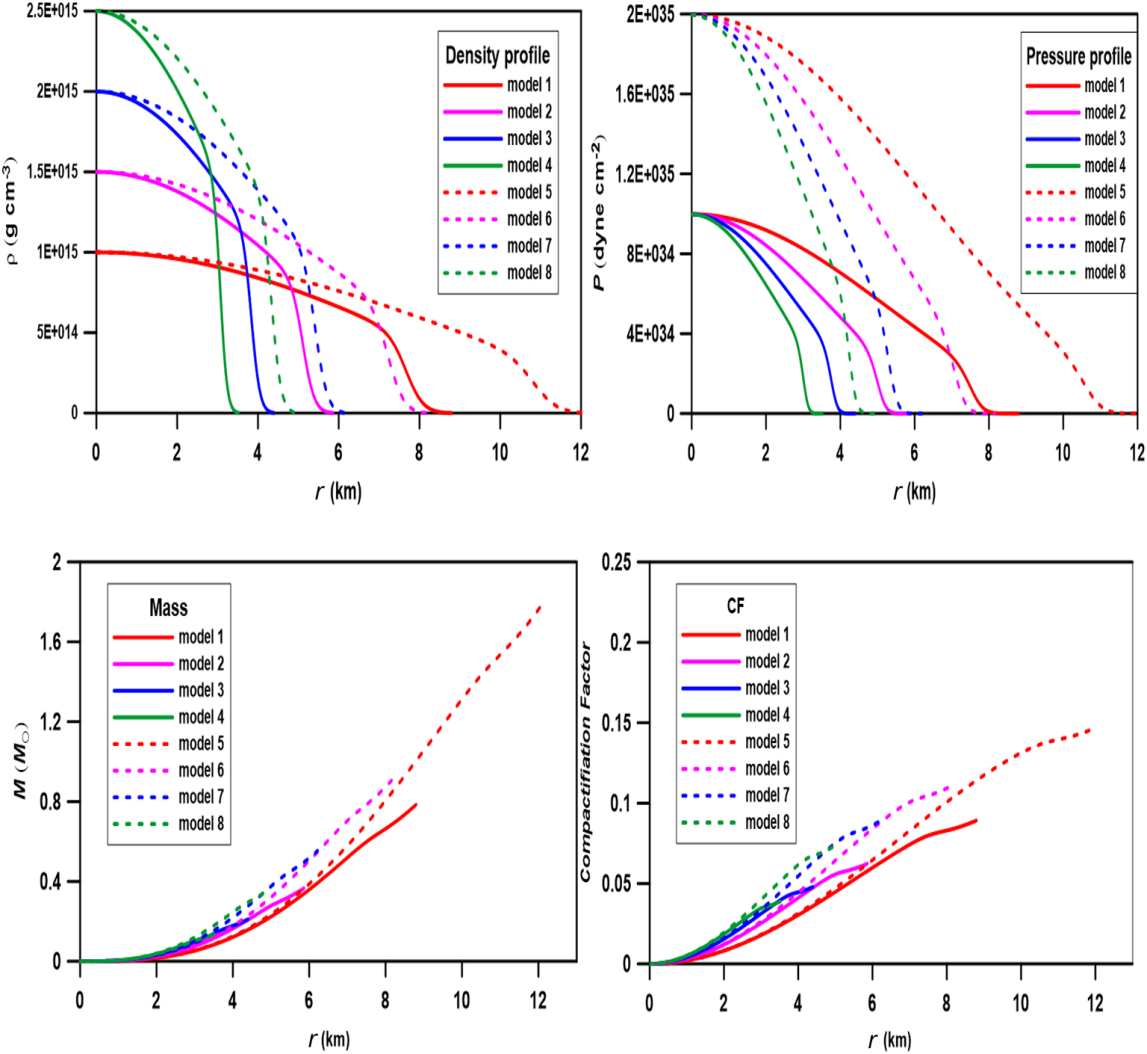
Density profiles, pressure profiles, mass-radius relations, and compactification factor for eight composite relativistic polytropic models computed for polytropic indexes from n = 1 to n = 2.

The results of calculations are plotted in Fig. 3 for the density profiles, pressure distributions, masses-radius relations, and the compactification factors (CF = m(r)/r). Comparisons between models with the same central densities but different central pressures (i.e., varying relativistic parameters), for example, model 1 and model 5 or model 2 and model 6, show that increasing the $\sigma$ lead to increase the radius of the star by about 30%. Also, as the $\sigma$ increases, the mass of the star increases. In Table 2, we list the CF calculated against the relativistic parameter $\sigma$, for any two models with the same central densities but different central pressures: the C.F. increases with increasing $\sigma$ (i.e., increasing *P*_*c*_). For instance, the C.F. of model 1 is about 60% of the CF of model 5 (the central pressure of model 5 is double that of model 1). Also, the CF of model 3 is about 50% of that of model 1 (the central density of model 3 is double that of model 1). The density and pressure profiles vary smoothly from the center toward the surface of the polytrope without discontinuity through the transition region (with width $\epsilon$) between n_1_ and n_2_. In the present calculations, we propose $\epsilon$ = 0.01 to allow a larger range of the variation of the polytropic index when crossing the transition region.

**Figure 4:**
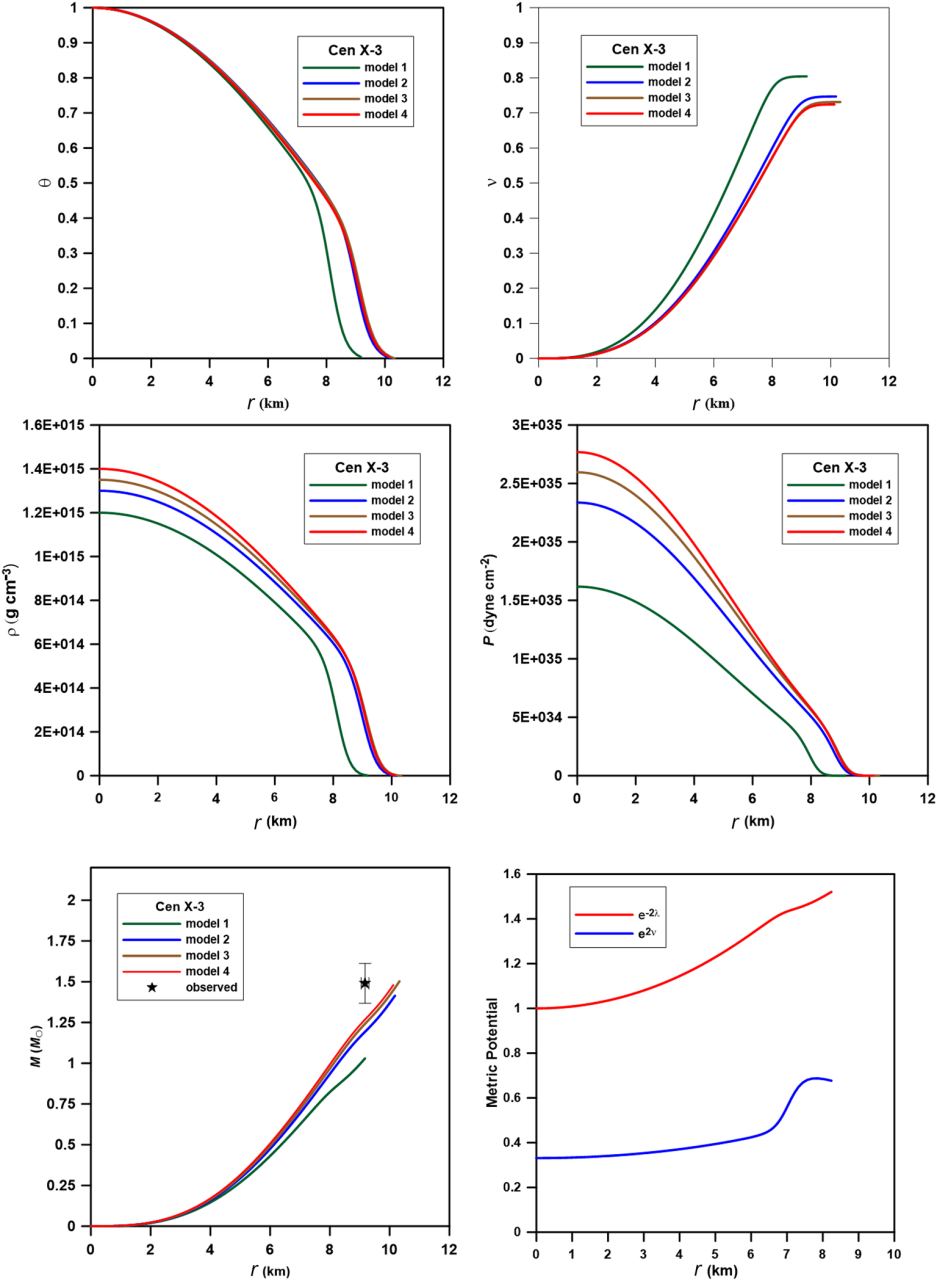
Variation of the Emden function (upper left), mass function (upper right), density (middle left), pressure (middle right), mass (bottom left), and the metric potential (bottom right) of the composite relativistic models for the star Cen X-3. The observed mass and radius are *M* = 1.49* M*_ʘ_ and *R* = 9.51 km.

**Figure 5:**
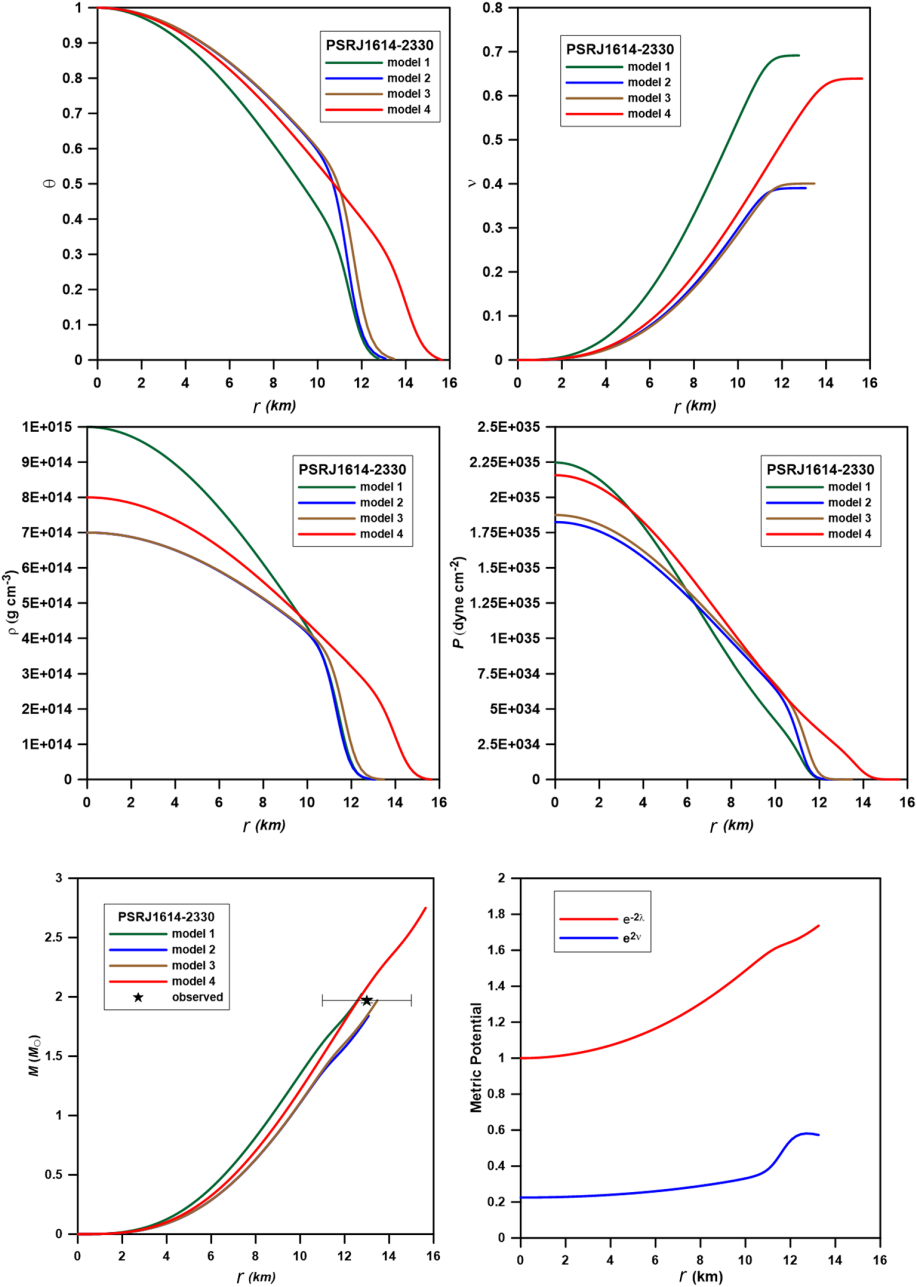
Variation of the Emden function (upper left), mass function (upper right), density (middle left), pressure (middle right), mass (bottom left), and the metric potential (bottom right) of the composite relativistic models for the star PSRJ1614-2330. The observed mass and radius are *M* = 1.97* M*_ʘ_ and *R* = 13 km.

**Figure 6:**
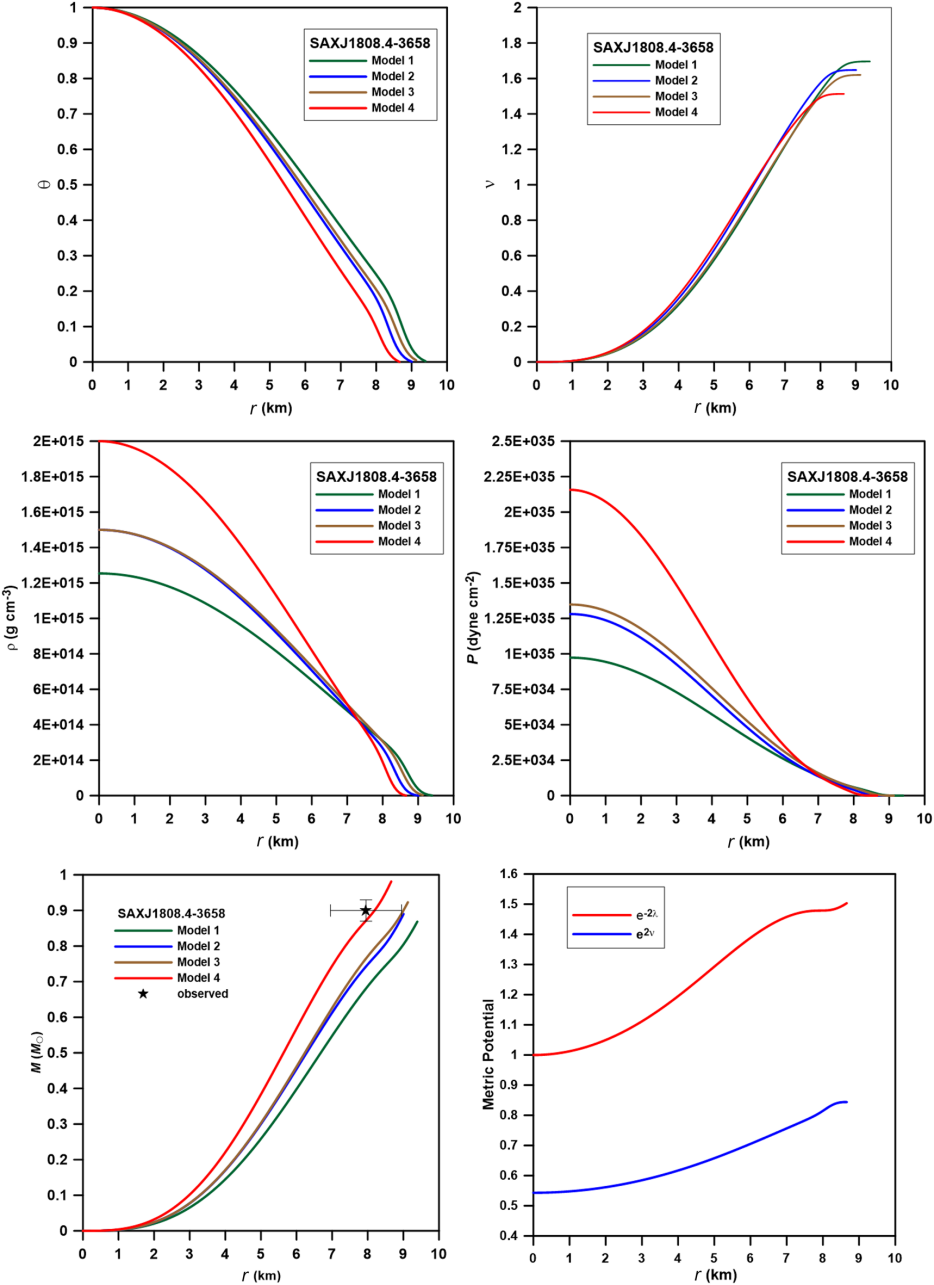
Variation of the Emden function (upper left), mass function (upper right), density (middle left), pressure (middle right), mass (bottom left), and the metric potential (bottom right) of the composite relativistic models for the star SAXJ1808.4-3658. The observed mass and radius are *M* = 0.9* M*_ʘ_ and *R* = 7.951 km.

To model the structure of the neutron star candidates Cen X-3 (mass 1.49 ± 0.49 *M*_ʘ_ and radius 9.51 ± 0.13 km^33^), PSR J1614-2230 (mass 1.97 ± 0.04 *M*_ʘ_ and radius 13 ± 2 km^34,35^), and SAXJ1808.4–3658 (mass 0.9 ± 0.3* M*_ʘ_ and radius 7.951 ± 1 km^36^), we computed four polytropic models for each star. Tables 3, 4 and 5 provide the relativistic parameter ($\sigma$), the central density ($\rho_{c}$), and the central pressure (*P*_c_) as input parameters to the code; the output parameters are the interface density($\rho_{i}$), the interface pressure (*P*_i_), the radius of the core (*R*_*c*_), the radius (*R*), and the mass (*M*). These computed physical quantities may be compared to the values of core-envelope models computed for n_1_ = 1 in the core and n_2_ = 2 in the crust for Cen X-3, PSRJ1614-2330, and SAXJ1808.4-3658^16,32^. In Figs. 4, 5 and 6, we plotted the variation of the Emden function (upper left), mass function (upper right), density (middle left), pressure (middle right), and mass (bottom) of the composite relativistic models for the three neutron stars. In what follows, we shall present results obtained for each star.

**Table 3:** Physical parameters of the polytropic models for Cen X-3.

Model	$\sigma$	$\rho_{c}$(10^15^)	*P*_*c*_ (10^35^)	$\rho_{i}$(10^15^)	*P*_*i*_ (10^35^)	*R*_*c*_ (km)	*R* (km)	*M* (*M*_ʘ_)
Model_Cen1	0.150	1.20	1.61784	0.7707	0.6787	5.889	9.17	1.03
Model_Cen2	0.20	1.30	2.33688	0.7818	0.8453	6.116	10.17	1.41
Model_Cen3	0.214	1.35	2.5958	0.7948	0.8994	6.117	10.39	1.50
Model_Cen4	0.220	1.40	2.7683	0.8162	0.9414	5.993	10.28	1.48

**Table 4:** Physical parameters of the polytropic models for PSR J1614-2230.

Model	$\sigma$	$\rho_{c}$(10^15^)	*P*_*c*_ (10^35^)	$\rho_{i}$(10^15^)	*P*_*i*_ (10^35^)	*R*_*c*_ (km)	*R* (km)	*M* (*M*_ʘ_)
Model_PSR1	0.250	1	2.2470	0.55799	0.69412	7.125	12.7701	2.02548
Model_PSR2	0.290	0.7	1.8245	0.4909	0.8975	9.170	13.076	1.8406
Model_PSR3	0.298	0.7	1.8749	0.4875	0.9093	9.380	13.469	1.9705
Model_PSR4	0.30	0.75	2.0223	0.3835	0.5288	8.259	16.1530	2.8407

**Table 5:** Physical parameters of the polytropic models for SAXJ1808.4-3658.

Model	$\sigma$	$\rho_{c}$(10^15^)	*P*_*c*_ (10^35^)	$\rho_{i}$(10^15^)	*P*_*i*_ (10^35^)	*R*_*c*_ (km)	*R* (km)	*M* (*M*_ʘ_)
Model_SAX1	0.086	1.25	0.9745	$0.4210$	$0.1097$	3.163	9.3920	0.8686
Model_SAX2	0.095	1.5	1.2807	$0.4816$	$0.1320$	2.891	9.0060	0.8902
Model_SAX3	0.10	1.5	1.3482	$0.4689$	$0.1317$	2.853	9.1277	0.9230
Model_SAX4	0.12	2	2.1571	$0.5596$	$0.1689$	2.422	8.6593	0.9813

Cen X-3, an occulting spectroscopic binary system member, is located in the galactic plane approximately 5.7 kiloparsecs away from the Carina-Sagittarius arm^37^. The visible component is Krzeminski's Star, a supergiant, whereas the X-ray is a revolving, magnetized neutron star. In Fig. 4, we plotted the density, pressure, and mass variation with the radius of the three models. In all models, the distributions of $\theta$,$\nu$, ρ, and *P* decreases smoothly from the center to the surface without discontinuity. The observed mass and radius values (*M* = 1.49 *M*_ʘ_, R = 9.51 km) fit approximately model_Cen4 with a total mass *M* = 1.48 M_ʘ_ and radius *R* = 10.28 km. Comparison of the interface range of density and pressure of our models ($\rho_{i}$ = 0.7707–0.8162 g cm^−3^, *P*_*i*_ = 0.6787–0.9414 dyne cm^−2^) for the star Cen X-3 and that from^28^ ($\rho_{i} = 1.2051 \times 10^{15}$ g cm^−3^ and $P_{i} = 1.977 \times 10^{35}$ dyne cm^−2^) gives smaller values of the interface density and pressure but a larger radius for the four models, that is simply because the core in our models represents about 60% of the total radius of the star while in^28^ the core occupied about 33% only.

PSR J1614-2230 is a millisecond pulsar in a binary system with a white dwarf in the Scorpius constellation. It spins on its axis around 317 times per second for a duration of 3.15 ms. It was discovered in 2006 using the Parkes telescope while studying unexplained gamma-ray sources in the Energetic Gamma Ray Experiment Telescope catalog^35^. The mass of PSR J1614-2230 (1.97 *M*_ʘ_) is the second greatest among all known neutron stars. A neutron star of such great mass limits the composition and structure of neutron stars, which are little known^36^. Our result for this star is listed in Table 4 and displayed in Fig. 5. The polytropic models predict that the core is about 60%-70% of the star's radius, so we found that the values of the density and pressure at the interface are smaller than those of the core-envelope model^34^. The observed mass and radius of the star are well fitted to model_PSR3 (the accepted model for PSR J1614-2230) with total mass *M* = 1.97 *M*_ʘ_ and radius *R* = 13.46 km, which are in good agreement with observations.

In 1998, the Italian-Dutch BeppoSAX spacecraft detected SAX J1808.4-3658, the first accreting millisecond pulsar. NASA's RXTE satellite observed X-ray pulsations at the 401 Hz neutron star spin frequency during an outburst in 1998^36^. A brown dwarf binary partner with a predicted mass of 0.05 solar masses orbits the neutron star every 2.01 h. SAX J1808.4-3658 has shown X-ray burst oscillations, quasi-periodic oscillations, and coherent X-ray pulsations, making it a key to understanding the timing behavior of low-mass X-ray binaries. The mass and radius from model_SAX4 (with total mass and radius 0.9813 M_ʘ_ and 8.6593 km) best fit the observed mass and radius (Fig. 6). The accepted model for this star predicts density and pressure at the center are as 2 × 10^15^ g cm^−3^ and 2.157 × 10^35^ dyne cm^−2^ higher than that predicted by^16^ and model_SAX1 (model_SAX1 is computed at the same values of the central density and pressure as^16^).

In Figs. 4, 5, and 6, we plotted the metric potentials $e^{2\nu (r)} {\text{ and }}e^{ - 2\lambda (r)}$ for the models proposed for the three stars. As is shown in the figures and demonstrated by Tooper^7^, the two metrics have to be well-defined at the center and regular as well as singularity-free throughout the interior of the star. The metric functions of the model satisfy $e^{ - 2\lambda (0)} = 1{\text{ and }}e^{2\nu (0)} = const$, i.e., finite at the center (r = 0) of the stellar configuration. Moreover, the derivatives of these potentials vanish at the center of the star, i.e., $(e^{2\nu } )^{\prime}_{r = 0} = (e^{ - 2\lambda } )^{\prime}_{r = 0} = 0$, which implies that the metric is regular at the center and well-behaved throughout the stellar interior. The component $g_{rr} = e^{ - 2\lambda }$ is always greater than or equal to unity, while the component $g_{rr} = e^{2\nu }$ is always less than unity and has a minimum at r = 0.

**Figure 7:**
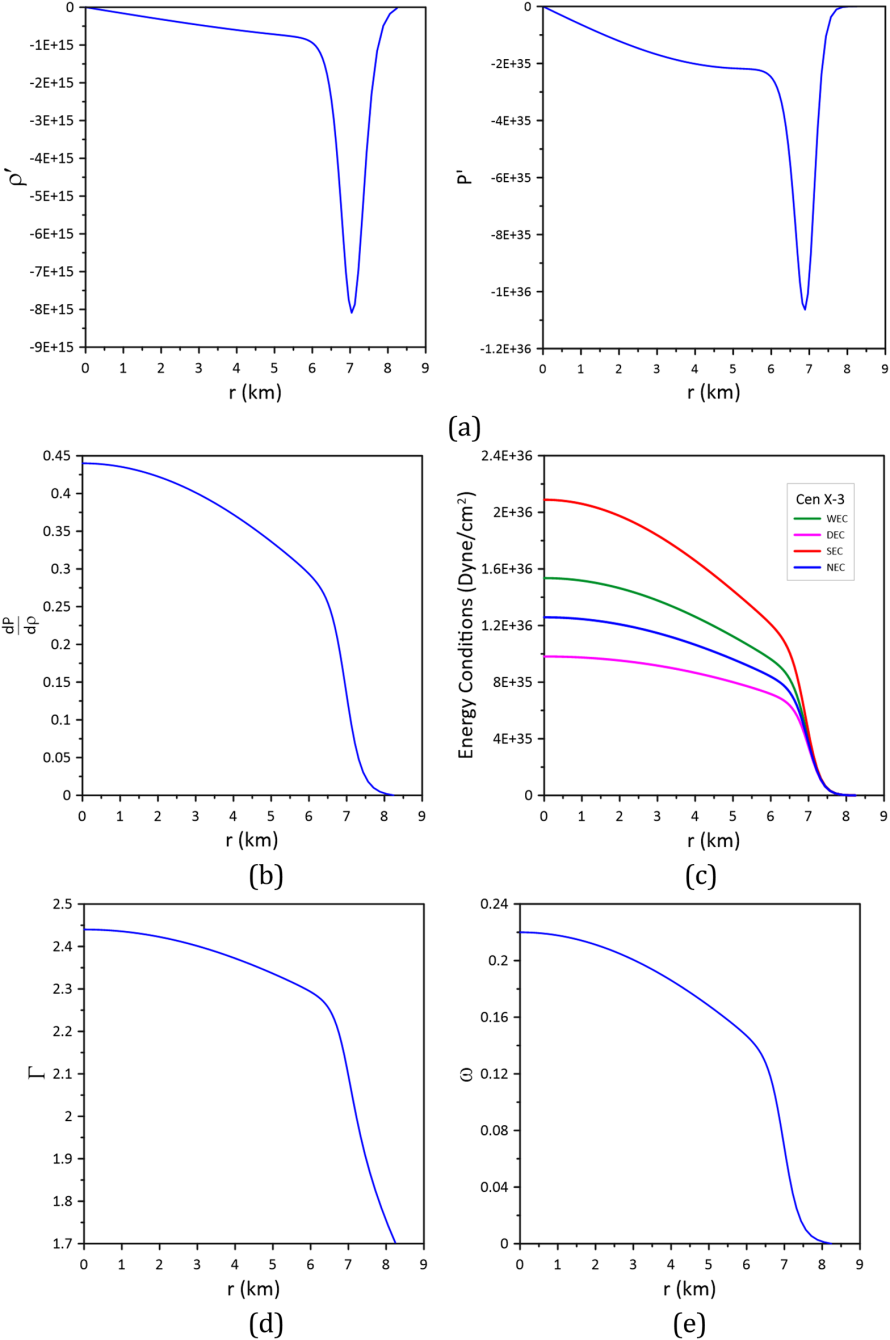
Stability of model_Cen4 (Table 3) proposed for modeling the star Cen X-3.

**Figure 8:**
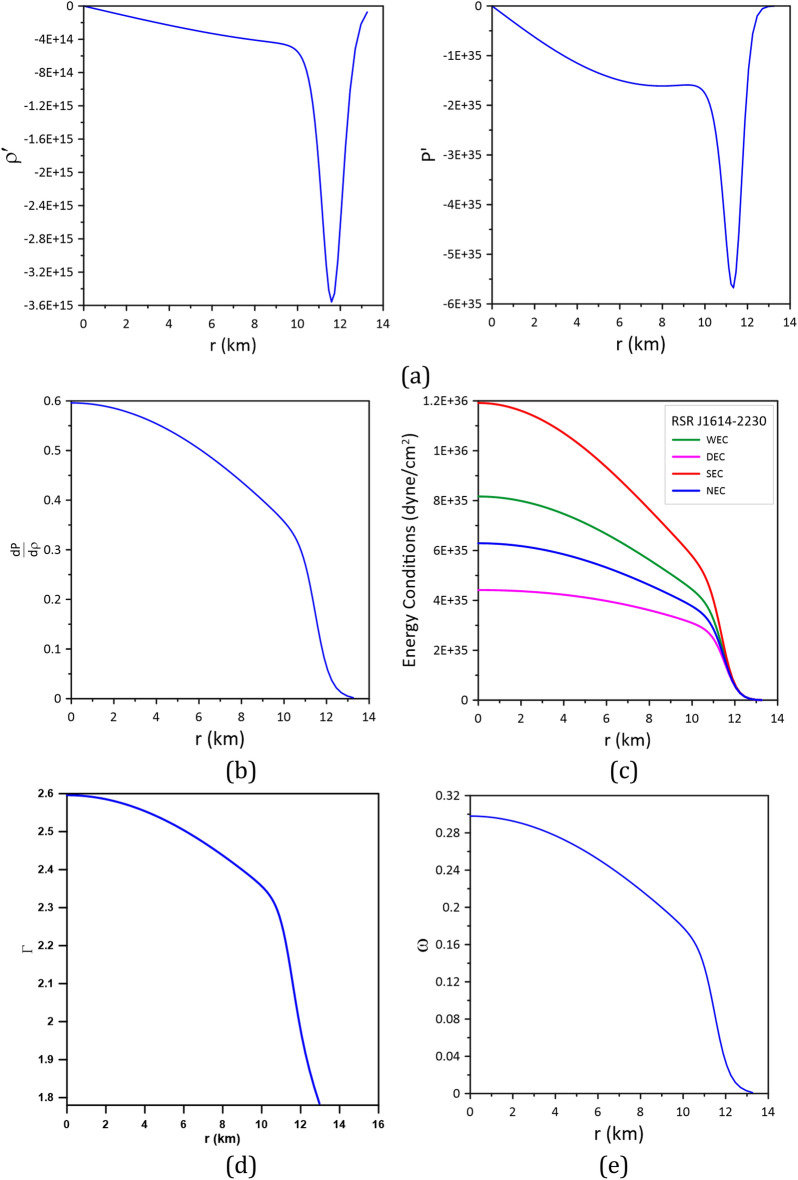
Stability of model_PSR3 (Table 4) proposed for modeling the star PSR J1614-22304.

To infer the stability of the accepted CTOV models for the three stars, the following conditions should be fulfilled^17,18^:

1. The density $\rho$ and the pressure $P$ should be positive, finite, and have regular behavior free from singularity within the stellar interior, i.e.,$\rho \ge 0, P\ge 0$. According to Figs. 4, 5 and 6, we can see that this condition is satisfied by the models.
2. The gradients of the density and pressure must be negative inside the star, i.e., $\frac{d\rho }{{dr}} \le 0{ , }\frac{dP}{{dr}} \le 0 \,$ and the pressure should vanish at the stellar boundary; these are shown graphically in Figs. 7a, 8a, and 9a.

**Figure 9:**
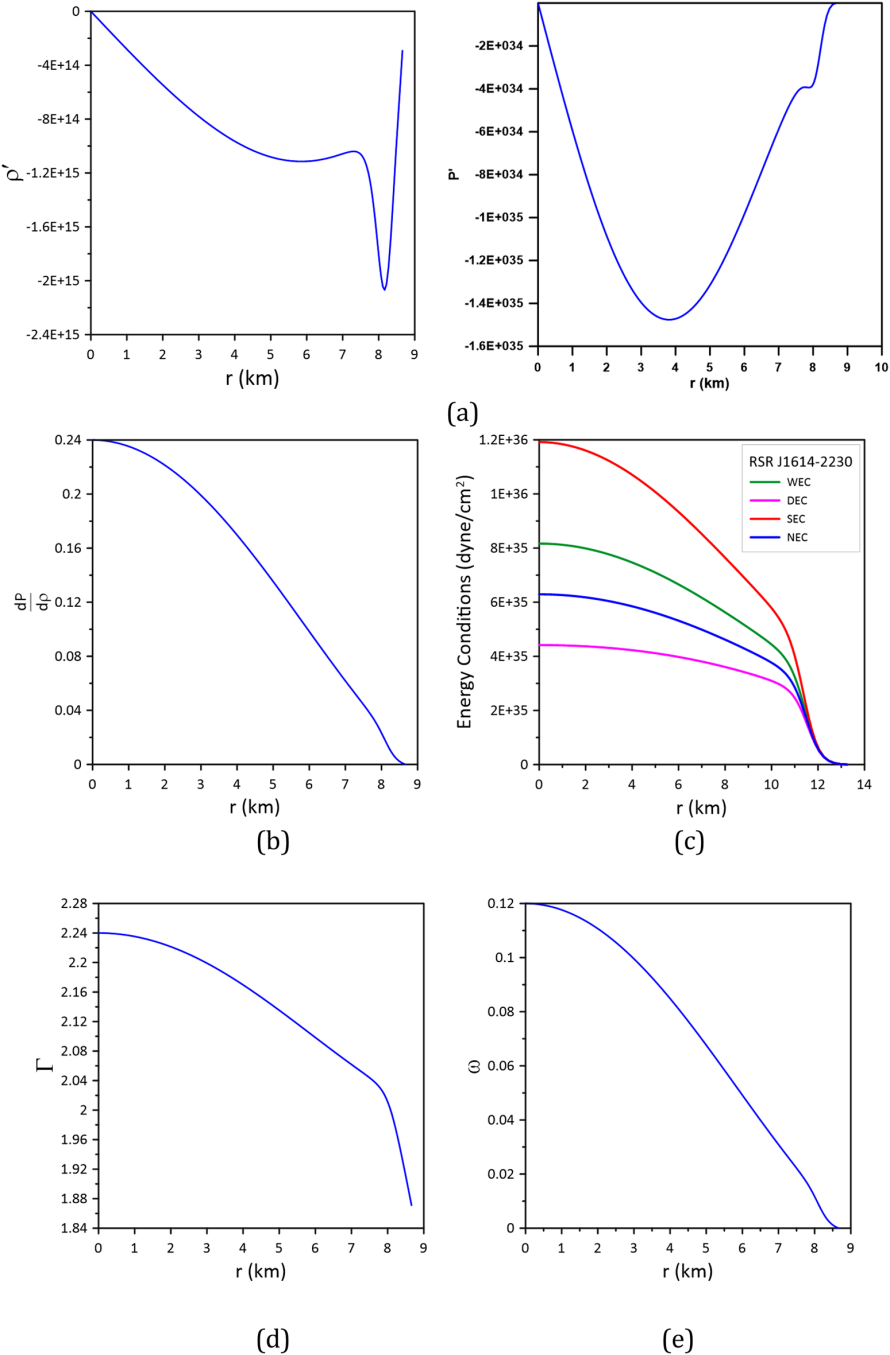
Stability of model_SAX1 (Table 5) proposed for modeling the star SAXJ1808.4-3658.
3. For stable stellar configurations, the speed of sound within the star must be less than the speed of light, i.e.,$0 \le \frac{dP}{{d\rho }} \le 1$. This is known as the causality condition, and it is satisfied by the model, see Figs. 7b, 8b, and 9b.
4. For an isotropic fluid sphere, the following energy conditions should be satisfied for stability.
  Null energy condition (NEC): $\rho \ge 0$.
  Weak energy condition (WEC): $\rho +P\ge 0$.
  Strong energy condition (SEC): $\rho +3 P\ge 0.$
  Dominant energy condition (DEC): $\rho -P\ge 0.$
  The models fulfill all these energy conditions see Figs. 7c, 8c, and 9c.
5. Stability via adiabatic index: To obtain a sable model for a relativistic isotropic fluid sphere, the adiabatic index $\Gamma$ should be greater than $4/3$^37^. The adiabatic index for isotropic stellar configuration is given by^38^
  $$\Gamma = \frac{\rho + P}{P}\frac{dP}{{d\rho }}$$.
  Figures 7d, 8d, and 9d show that the adiabatic index is greater than 4/3 throughout the stellar interior.
6. For the matter within the star to be non-exotic, the value of the EoS parameter $\omega$ should be positive and must satisfy Zeldovich’s condition, $0 \le \omega = \frac{P}{\rho } \le 1$, at the stellar center^39^. The models fulfill this condition; see Figs. 7e, 8e, and 9e.

## Conclusions

By simultaneously solving the polytropic equation of state and Einstein field equations, this work offered innovative composite polytropic models for compact stars. Using a non-uniform polytropic index, we obtained the Tolman–Oppenheimer–Volkoff equation for the relativistic composite polytrope (CTOV). We used numerical integration to solve the CTOV equation. We estimated the mass and Emden functions for various relativistic parameters and polytropic indices appropriate for neutron stars to examine the star's structure. When we test the computational code by comparing composite Newtonian models with those published in the literature, we find good agreement because the computation results demonstrate that, as the relativistic parameter approaches zero, we recover the well-known Lane-Emden equation from the Newtonian theory of polytropic stars.

Additionally, there was good agreement between the relativistic model of the single polytrope estimated from the TOV and that computed from the CTOV. From the star's center to its surface, the distributions of the mass function, the Emden function, the density, the pressure, and the mass exhibit smooth variation. One advantage of the computed CTOV models is that they are multi-layered, not double-layered.

Using observable values of the mass and radius of the pulsars Cen X-3, PSR J1614-22304, and SAXJ1808.4-3658 and with central density and pressure compatible with neutron core pulsars, we verified the physical correctness of the model. We used central density and pressure parameters similar to those in the literature to construct four models for each star so that they could be compared. According to the accepted models for Cen X-3 and PSR J1614-22304, the core radius of the star is estimated to be between 50 and 60% of its overall radius. We found that the core radius of star SAXJ1808.4-3658 is around 30% of the overall radius. We computed the masses and radii of the three stars as follows: *M *= 1.48 *M*_ʘ_ and *R *= 10.28 km for Cen X-3, *M *= 1.97 *M*_ʘ_ and *R *= 13.46 km for PSR J1614-2230, and *M *= 0.98 *M*_ʘ_ and *R *= 8.65 km for SAXJ1808.4-3658. Taking into account the errors in observation, the computed masses and radius are in good agreement for the three investigated stars.

The metric potentials for the models proposed for the three stars are found to be well-defined at the center and regular as well as singularity-free throughout the interior of the star. It can be confirmed that every single physical parameter, stability condition, and energy condition inside the stars are on a sustainable trend and vary smoothly without interruption.

Using a single EoS may not be the best option for realistic modeling of the entire star^22,40^. For example, using composite EoS instead of single EOS^41^, used polytropic equations to simulate the EoS in each location in the star: the crust, the outer core, and the inner core; four polytropes are used in the crust and three in the outer and inner cores. Then, a piecewise function with seven polytropes represents the whole EoS.

Conversely, more electromagnetic and gravitational-wave measurements are being made of neutron stars. These measurements enable us to restrict the dense matter equation of state and comprehend the fundamental processes inside these small objects. The TOV equations may be reversed using these accurate observations to get the EOS for neutron stars with global parameters like mass and radius. Many techniques are implemented to obtain EOS from the observed mass-radius relation of neutron stars; examples of these techniques are^42–44^. To obtain a homogenous sample of the CTOV models, we can calculate the grid of models covering an extensive range of the central density and relativistic parameters and compare the computed mass-radius relation with the observations. Consequently, the CTOV models for neutron stars may be compared to models calculated based on non-analytical EOS; this will be done in future work.

## Data Availability

The datasets used and/or analysed during the current study are available from the corresponding author upon reasonable request.
